# A rapid, high-volume cervical screening project using self-sampling and isothermal PCR HPV testing

**DOI:** 10.1186/s13027-020-00329-0

**Published:** 2020-10-22

**Authors:** Andrew Goldstein, Yang Lei, Lena Goldstein, Amelia Goldstein, Qiao Xu Bai, Juan Felix, Roberta Lipson, Maria Demarco, Mark Schiffman, Didem Egemen, Kanan T. Desai, Sarah Bedell, Janet Gersten, Gail Goldstein, Karen O’Keefe, Casey O’Keefe, Tierney O’Keefe, Cathy Sebag, Lior Lobel, Anna Zhao, Yan Ling Lu

**Affiliations:** 1 Centers for Vulvovaginal Disease, Washington, DC USA; 2 Peking University Cancer Hospital, Beijing, China; 3 Yale University, New Haven, CT USA; 4 Duke University, Durham, NC USA; 5 United Family Hospitals, Beijing, China; 6 Medical College of Wisconsin, Milwaukee, WI USA; 7 Division of Cancer Epidemiology and Genetics, National Cancer Institute, National Institutes of Health, Rockville, MD USA; 8 New Age Women’s Health, Miami, Florida, USA; 9 Annapolis Dermatology Center, Annapolis, MD USA; 10 Bellingham Bay Family Medicine, Bellingham, WA USA; 11 Pacific Northwest Urology Specialists, Bellingham, WA USA; 12 Scripps College, Claremont, California, USA; 13 MobileODT, Tel Aviv, Israel

## Abstract

**Objective:**

Rapid, high-volume screening programs are needed as part of cervical cancer prevention in China.

**Methods:**

In a 5-day screening project in Inner Mongolia, 3345 women volunteered following a community awareness campaign, and self-swabbed to permit rapid HPV testing. Two AmpFire™ HPV detection systems (Atila Biosystems) were sufficient to provide pooled 15-HPV type data within an hour. HPV+ patients had same-day digital colposcopy (DC) performed by 1 of 6 physicians, using the EVA™ system (MobileODT). Digital images were obtained and, after biopsy of suspected lesions for later confirmatory diagnosis, women were treated immediately based on colposcopic impression. Suspected low- grade lesions were offered treatment with thermal ablation (Wisap), and suspected high-grade lesions were treated with LLETZ.

**Results:**

Of 3345 women screened, 624 (18.7%) were HPV+. Of these, 88.5% HPV+ women underwent same-day colposcopy and 78 were treated. Later consensus histology results obtained on 197 women indicated 20 CIN2+, of whom 15 were detected and treated/referred at screening (10 by thermal ablation, 4 by LLETZ, 1 by referral).

**Conclusions:**

Global control of cervical cancer will require both vaccination and screening of a huge number of women. This study illustrates a cervical screening strategy that can be used to screen-and-treat large numbers of women. HPV self-sampling facilitates high-volume screening. Specimens can be tested rapidly, promoting minimal loss-to-follow-up. Specifically, the AmpFire™ system used in this study is highly portable, simple, rapid (92 specimens per 65 min per unit), and economical. Visual triage can be performed on HPV+ women with a portable digital colposcope that provides magnification, lighting, and a recorded image. Diagnosis and appropriate treatment remain the most subjective elements. The digital image is under study for deep-learning based automated evaluation that could assist the management decision, either by itself or combined with HPV typing.

## Introduction

Infection with human papillomavirus (HPV) is a leading cause of cancer among women worldwide with approximately 500,000 new cervical cancer cases and 250,000 deaths each year [1]. Cervical cancer is caused by persistent infection with a group of carcinogenic HPV genotypes (HPV16, 18, 31, 33, 35, 39, 45, 51, 52, 56, 58, 59, and probably HPV68) [2]. The importance of cervical cancer is accentuated by the relatively young average age at incidence and death.

Cervical cancer screening strategies have evolved from cytology-based to HPV-based [3]. Following the identification of HPV as the cause of cervical cancer and the development of sensitive HPV tests, HPV-based screening permits the extension of screening intervals and increased impact per number of lifetime screens [3].

Without compromising yield of disease, self-sampling can increase participation and reach of cervical cancer screening programs. Self-sampling is comparable to clinician-obtained sampling for HPV-based screening, is well accepted in many populations, and has been incorporated into screening programs to improve coverage [4, 5].

Currently, there are no national cervical cancer screening programs in China and only 10–30% of Chinese women report having ever had cervical cancer screening [6]. Although incidence and mortality rates (15.3/100,000 and 4.6/100,000, respectively) are moderately high, the population is so large that every year, there are approximately 100,000 new cervical cancer cases and 30,000 deaths in China [7]. In general, women living in rural areas are less likely to report ever having had cervical cancer screening and mortality rates from cervical cancer are up to 48% higher [6, 8–10].

The Inner Mongolia Minority Autonomous Region (Inner Mongolia), is a vast territory that stretches in a great crescent for 1500 miles across northern China. Inner Mongolia was part of the ancient Silk Road region and is bordered to the north by Mongolia and Russia. Inner Mongolia is a geographically diverse and relatively underdeveloped province, with a population of over 24 million in the 2010 census. Forty-nine ethnic groups live in Inner Mongolia though the majority are Mongolian or Han. Additional ethnicities include Manchu, Hui, Daur, Ewenki, Oroqen, and Korean. More than 58% of the population lives in rural areas.

Due to the level of development, complex geography, and dispersed population across the rural parts of the majority of Inner Mongolia, the conventional, multi-step screening process for cervical cancer is not feasible. The traditional process of screening with cytology, colposcopy, biopsy and subsequent treatment of women diagnosed with cervical precancer is too resource and expertise-intensive for low-income, vast regions such as Inner Mongolia. Accordingly, techniques such as visualization with acetic acid (VIA) and HPV testing have been studied as alternative methods.

The prevalence of HPV varies amongst different ethnic and geographic regions [11, 12]. In China, the prevalence and genotype distributions of HPV are well documented. HPV genotypes 16, 18, 52 and 58 are the most common cancer-causing types amongst Chinese women, with differing distribution rates throughout the country. The prevalence of high-risk HPV within Inner Mongolia ranges from 14.5–36.0%, and this varies significantly between different ethnicities [13–15].

This study demonstrates the feasibility of a rapid, high-volume screening approach that combines self-sampled HPV testing and digital colposcopy triage to reach unscreened populations, as exemplified by Inner Mongolia, China.

## Methods

**Fig. 1:**
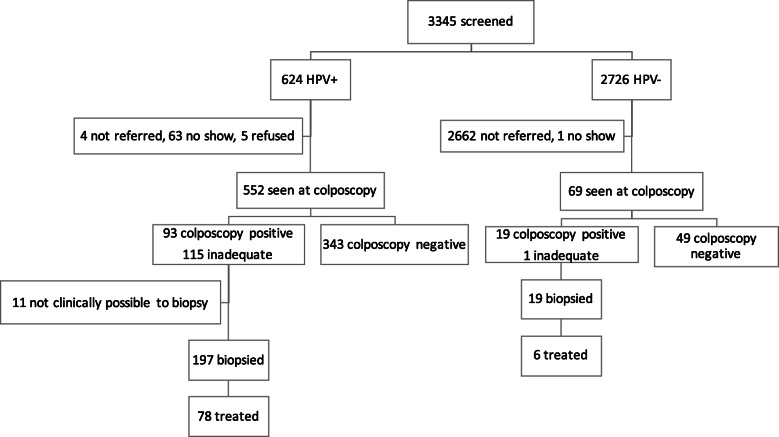
Study population

This is a cross-sectional analysis using data from women attending a same-day, high-volume screening demonstration in Inner Mongolia, China. Women were contacted and, following an awareness campaign, 3345 agreed to participate and provided consent (Fig. 1). Participants were female, 30–65 years old, who provided consent to participate in the study. We excluded women who: knew or thought they might be pregnant, were unable to provide informed consent, were seriously ill, had a gross cervical mass, history of previous treatment for cervical cancer, or complete hysterectomy, or had cervical cancer screening in the past 5 years.

During a 6-day period in May 2019 that consisted of 4 clinic days and 2 travel days, 3345 Chinese women aged 30 to 65 were screened in three different medical clinics in the Inner Mongolia Minority Autonomous Region. IRB approval was obtained from United Family Hospitals Investigational Review Board, Beijing, China. Local health officials notified and registered potential participants in the preceding weeks. The vast majority of women had never been screened for cervical cancer. After obtaining informed consent, participants received a brief explanation of HPV and cervical cancer via a pre-recorded video, as well as instructions on how to obtain a self-sampled vaginal specimen.

**Fig. 2:**
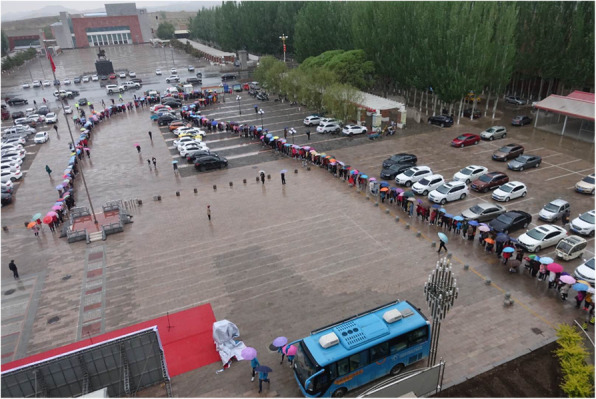
Health camp approach in the Inner Mongolia Minority Autonomous Region, Photo acknowledgement: Adam Qin

We screened 460–990 women per day (Fig. 2). The first 79 arrivals were referred directly to colposcopy after self-sampling, without awaiting the test results, to make best use of the physicians’ time. This provided a subset of 69 HPV-negative referrals. Subsequently, only HPV-positive women were referred for colposcopic examination.

Self-collected vaginal specimens were collected using dry brushes and tested for 15 high-risk HPV (HPV16, HPV18, or a pool of 13 types including HPV31, 33, 35, 39, 45, 51, 52, 53, 56, 58, 59, 66, 68) on site using two AmpFire™ (Atila Biosystems, California) machines running concurrently. While waiting for test results, women participated in a joint breast cancer screening effort. All women positive for high-risk HPV (hrHPV+) results were contacted via text message and returned for colposcopic examination the same day or the following day.

Digital Colposcopy (DC) was performed with the EVA system 90 s after the application of acetic acid. All DC was performed by one of six physicians and the EVA system was used to obtain 1–3 DC images per patient. Thin acetowhite lesions were considered suspicious for CIN1. Thick acetowhite lesions, rapidly appearing lesions, lesions with course mosaicism or punctuation, and lesions with sharp borders were considered suspicious for CIN2 + .[27] The limitations of visual assessment for grading are acknowledged and discussed below.

If DC was positive for cervical abnormalities (suspected CIN1+), patients underwent cervical biopsy of the exocervix using a SoftBiopsy™ (Histologics, California) brush for subsequent confirmatory diagnosis, but were treated presumptively with thermocoagulation or loop electrosurgical excision procedure (LLETZ) the same day. The SoftBiopsy™ (Histologics, California) brush obtains tissue from the entire exocervix to be processed in a single slide. Compared with multiple biopsies from biopsy forceps, it is faster to obtain tissue from multiple areas of the exocervix and it causes less trauma and bleeding, making it easier to perform thermocoagulation (if needed) immediately after biopsy.

Thermocoagulation was performed when DC findings were suspicious for CIN1 lesions using one of two different thermocoagulation systems: C3 thermo-coagulator (WISAP, Germany) or TC thermocoagulator™ (Cure Medical, Utah). A LLETZ was performed when findings were highly suspicious for CIN2+ or when thermal ablation was not technically possible.

If no acetowhite changes were seen and the entire transformation zone was visible, the patients were informed of the findings and not immediately treated. Repeat HPV testing in 1 year was advised; although the availability of such follow-up was recognized not to be assured, immediate treatment in the absence of a visible lesion was judged not to be warranted. If the transformation zone was not fully visible on DC or if the lesion extended in the endocervical canal, endocervical curettage (ECC) was performed.

Overall, at colposcopy, clinician colposcopic impression led to 216 women biopsied and, of those, 84 immediately treated. Immediate treatment included thermal ablation, LLETZ, and none (including women who refused treatment and those treated later, based on histologic results). All biopsy specimens were subsequently processed, and read twice, by two pathologists (QXB, JF). The original histopathologic diagnoses included CIN1, CIN1–2, CIN2, CIN2–3, CIN3, and inadequate. For this research analysis, precancer case status among the HPV-positive women was defined as follows: CIN3 by either pathologist or CIN2 by both pathologists. The remaining adequate samples were considered <CIN2.

The few women with unanticipated high-grade lesions (CIN2+) were later contacted with their histology results and were counseled to follow up at the regional hospital for appropriate management. As mentioned, while women were waiting for the results of their HPV tests, they were screened for breast cancer with a portable ultrasound unit (results reported separately). Throughout the screening and treatment process, for educational purposes, local doctors and nurses were taught colposcopy techniques by the visiting physicians and received training to evaluate DC images.

## Results

Using self-sampled HPV testing, 3345 women were screened. Most women screened were ethnically Han (64.4%) and Meng (20.1%) (Table 1). The majority (63.7%) were ages 35–49.

**Table 1:** General information of the screened residences in three areas in Inner Mongolia

	Areas			
	Balinyouqi	Aershan	Molidawa	Total
Age				
30 ~ 34	76 (8.3)	5 (2.6)	55 (5.5)	136 (6.4)
35 ~ 39	143 (15.7)	15 (7.8)	235 (23.4)	393 (18.5)
40 ~ 44	157 (17.2)	32 (16.6)	237 (23.6)	426 (20)
45 ~ 49	222 (24.3)	46 (23.8)	268 (26.7)	536 (25.2)
50 ~ 54	153 (16.8)	60 (31.1)	152 (15.1)	365 (17.2)
55 ~ 59	116 (12.7)	31 (16.1)	44 (4.4)	191 (9)
60 ~ 64	45 (4.9)	4 (2.1)	14 (1.4)	63 (3)
Mean	46.2 ± 8.0	48.4 ± 6.2	44.0 ± 6.7	45.4 ± 7.4
Ethnicity				
Han	540 (59.1)	148 (75.1)	681 (67)	1369 (64.4)
Meng	344 (37.7)	39 (19.8)	44 (4.3)	427 (20.1)
Da	0 (0)	0 (0)	189 (18.6)	189 (8.5)
unknown	29 (3.2)	10 (5.1)	103 (10.1)	139 (6.3)
Parity				
0	4 (0.4)	3 (1.6)	13 (1.3)	20 (0.9)
1	393 (43.6)	157 (84)	553 (55)	1103 (51.9)
2	442 (49.1)	22 (11.8)	376 (37.4)	840 (39.5)
≥ 3	60 (6.7)	5 (2.6)	63 (6.3)	128 (6.1)
unknown	2 (0.2)	0 (0)	0 (0)	2 (0.1)

HPV-positive women (624, 18.7%) were invited for colposcopy and 552 attended the medical examination. Colposcopic impression was inadequate for 20.8%. Of the rest, 78.5% impressions were normal and not requiring biopsy.

The characteristics of the 197 HPV-positive women that underwent colposcopy and had a biopsy are listed in Table 2. Biopsies were collected with SoftBiopsy® brush (44.2%), ECC (52.8%), or both (3%).

**Table 2:** Descriptive statistics for HPV-positive women with biopsies taken (*n* = 197)

	Frequency n (%)
HPV results	
16^a^	33 (17.2)
18	16 (8.3)
Others	143 (74.5)
.	5
Colposcopy impression	
High	21 (10.7)
Low	72 (36.5)
Inadequate	104 (52.8)
Biopsy taken	
Brush	87 (44.2)
ECC	104 (52.8)
Both	6 (3.0)
Histology	
CIN2+	20 (10.2)
< CIN2	176 (89.8)
Treatment	
Thermoablation	70 (89.7)
LLETZ	7 (9.0)
Referral	1 (1.3)
None	119

^a^ Includes 1 multiple infection with HPV types 16 and 18

From the 78 women who received treatment, 71 (89.7%) were treated with thermal ablation and 7 (9.0%) with LLETZ.

This rapid, high-volume program yielded 0.60% of precancer (20 out of 3345) in the screening population. From the 20 cases identified by histology, 10 were infected with HPV16, 1 with HPV18, and 8 with other types (HPV status was unknown for 1 case) (Table 3). Thus, we observed the expected increased risk associated with HPV16 and HPV18. Fifteen of the 20 HPV-positive cases received treatment: 10 by thermal ablation, 4 by LLETZ, 1 by referral. We notified the clinicians of the 5 untreated cases for follow-up after the program ended.

**Table 3:** Histology by HPV status for women with biopsies taken (*n* = 216)

HPV status	Histology		
	CIN2+ n (col %)	<CIN2 n (col%)	Total n (col%)
16+	10 (50.0%)	23 (11.7%)	33^a^ (15.3%)
18+	1 (5.0%)	15 (7.7%)	16 (7.4%)
Other hrHPV+	8 (40.0%)	135 (68.9%)	143 (66.2%)
Unknown type	1 (5.0%)	4 (2.0%)	5 (2.3%)
HPV- (controls)	0 (0.0%)	19 (9.7%)	19 (8.8%)
Total	20	196	216

^a^ Includes 1 multiple infection with HPV types 16 and 18

## Discussion

As its main conclusion, this study showed that large-scale cervical cancer screening efforts are feasible using self-sampling and rapid HPV testing. Availability of results within 2 h of collection made it possible for a single-visit screen and treat program.

Among the 20 CIN2+ cases, we saw the expected strong relationship between HPV positivity and presence of precancer, with some insights on partial HPV genotyping. However, our evaluation of absolute sensitivity of the HPV test was imperfect, because most HPV-negative women did not receive colposcopic evaluations. HPV DNA negativity typically predicts an extremely low risk of prevalent or incipient cervical cancer (or even precancer) [16].

As the success of cervical cancer screening programs has dramatically decreased the rates of cervical cancer in developed nations, the global burden of this disease falls mainly in areas of limited resources. It is estimated that the number of 35–64-year-old women in rural China exceeds 150 million. As the vast majority of these women have never been screened, strategies must be developed that allow for the screening and treating of large numbers of women. We demonstrate a screen-and-treat model that is low-cost, rapid, and capable of being implemented on a large scale, as evidenced by the ability to screen 3345 women in less than one week. The authors have previously described a similar strategy for cervical cancer screening in the Yunnan Province [17].

The first component of this model consists of self-obtained HPV specimens for rapid testing. It is practical for women in China to obtain self-collected HPV specimens, which then undergo rapid testing. Self-swabbing for HPV appears to be acceptable to Chinese women, regardless of their ethnicity.

Rapid testing is a critical aspect of this model as it allows for same-day treatment. The highly portable isothermal PCR based HPV AmpFire™ testing system takes 1 h to run 94 specimens and provides partial genotyping (another test kit is available for complete typing). It is minimally labor intensive and does not require a high degree of technical expertise to run. The self-swab specimens can be stored dry (without collection media) and the reagents can be stored at room temperature for several weeks. Furthermore, the price per specimen is approximately $US 7 and the same system can be used to test for other sexually transmitted infections.

The second component of this proposed model is to perform DC on all HPV+ women with a highly portable DC system. Our program included 6 physicians, a level of expert involvement that is often not available. Given the huge number of women in China who have never been screened as compared to the number of physicians available, DC is a modality that could be performed (if authorized) by mid-level providers such as nurses or midwives. Digital images obtained have excellent resolution and areas of question can be magnified for better interpretation. In addition, captured images can be used for continued education of mid-level providers, and the images can be sent electronically, through a secure, cloud-based portal, to an expert colposcopist for consultation of difficult cases.

Our study supports assistive technology to aid clinical decision making based on human visual impression. Even when performed by experienced specialists, colposcopic impression shows subjectivity and limited reliability or inter-observer agreement [18]. In addition to its inherent subjectivity, it requires highly trained human resources that are not available in some settings.

Ongoing efforts using Automated Evaluation (AVE) of the cervix show promising results using archived images from a consortium of sites [19, 20]. AVE has been evaluated for cervical cancer screening in the general population and as triage of HPV positive tests. When used as an aid to VIA (possibly in combination with HPV type), it might improve risk stratification of screening and triage, minimizing the subjectivity of human visual interpretation.

The third component of this screen-and-treat model is to use the clinical impression obtained from the DC images, perhaps assisted by AVE, to determine immediate and appropriate treatment. Thermocoagulation is inexpensive, highly portable, and a highly effective treatment modality for cervical intraepithelial lesions caused by HPV.

Cervical cancer screening programs could be combined with same-day vaccination efforts for HPV and other medical interventions. For example, we combined cervical and breast screening as part of the same program.

The screening model proven to be feasible by this effort is promising, but might not be widely applicable at the present time. The health camp approach (Fig. 2) is, by its nature, crowded and might be contraindicated until social gathering is again safe. Where social distancing remains a priority due to COVID-19, cervical cancer prevention efforts will need to be adjusted accordingly to maintain a net benefit for participants, as discussed by Ajenifuja et al. in a companion article. Portability of this screening model in terms of feasibility, acceptability, yield, sustainability, overall cost-effectiveness, and long-term impact also needs to be demonstrated in diverse geographic settings.

## Data Availability

Data available upon reasonable request from corresponding author Dr. A. Goldstein.
